# A Word to the Wise

**Published:** 1883-08

**Authors:** 


### A WORD TO THE WISE.

We call the attention of our professional brethren of the State to the suggestions of Dr. Gaither on another page. The facts he gives of the peculiar difficulties, which have, ever since the war, surrounded and trammeled the country practice—difficulties, which so far from being lessened as the years passed away, have only been augmented and intensified—are well worth a more serious consideration of the State Association.

First, as regards members, the laborious, self-sacrificing practitioner of the country and small towns, are like the rank and file of the army, the backbone of the service, the very hope of the Association. But second, as regards real ability, skill and high moral worth, many of these gentlemen are not a whit behind the most reputable of the city brethren, who, may only be more bespattered with printers' ink:

We have now in our mind's eye an earnest, conscientious, over-worked physician of the country, not a hundred miles from the capitol in Atlanta—a man of the highest social worth and of the most approved skill, both in surgery and medicine—a man who pursued on scanty means his professional studies in the best schools of Europe and America, a man who, all his professional life, has scorned to use the little, easy, but potent means by which so many inferior men are hoisted and drummed to fame; a man, who, with the enthusiasm of the true patriot that he is, brought his professional ability a free will offering to the South in her recent trouble, under the leadership of the gallant Hampton—and every soldier knows what that cavalry service was—served with distinction as a surgeon from the fall of Sumter to the surrender at Appomattox

And now battle and life worn but still pursuing with much of the ardor of youth, his mission of care, having a large family to maintain, and surrounded by a community to whom the fortunes of war and reconstruction have left little else than strong arms and stout hearts, he is often debarred by sheer lack of means, making no mention of other obstacles referred to by Dr. Gaither, from attendance upon the regular sessions of the Association.

It will not be out of place to add that the dress in which he appears in his daily rounds, and even in country gatherings, would in the eyes of the average medical dude of the city, consign him to a place among the nobodies of his honorable profession.

